# Association between SOD2 V16A variant and urological cancer risk

**DOI:** 10.18632/aging.102658

**Published:** 2020-01-12

**Authors:** Li-Feng Zhang, Kai Xu, Bo-Wen Tang, Wei Zhang, Wei Yuan, Chuang Yue, Li Shi, Yuan-Yuan Mi, Li Zuo, Li-Jie Zhu

**Affiliations:** 1 Department of Urology, The Affiliated Changzhou No.2 People's Hospital of Nanjing Medical University, Changzhou 213003, China; 2 Department of Oncology, Taizhou People's Hospital, Taizhou 225300, China; 3 Department of Cardiology, Taizhou People's Hospital, Taizhou 225300, China; 4 Department of Urology, Affiliated Hospital of Jiangnan University, Wuxi 214000, China

**Keywords:** SOD2, variant, urological cancer, *in silico*, analysis

## Abstract

Background: The correlation between superoxide dismutase 2 (SOD2) V16A variant and urological cancer susceptibility has been widely studied, however, with divergent results.

Results: Totally, 9,910 cancer patients and 11,239 control subjects were enrolled. V16A variant is associated with an increased susceptibility to urological cancer (A-allele vs. V-allele: OR = 1.06, 95% CI = 1.00 – 1.13, *P* = 0.047; AA+AV vs. VV: OR = 1.09, 95% CI = 1.02 – 1.16, *P* = 0.008), especially for prostate cancer (PCa). Serum SOD2 level of PCa patients with VV+VA genotypes was lower than in those with AA genotypes. SOD2 expression is downregulated in both prostate and bladder cancer, as compared to the control. Furthermore, SOD2 was found to be downregulated in more advanced PCa participants, as compared to the ones in early stages. PCa subjects with low SOD2 expression displayed a shorter disease-free survival (DFS) time compared to that of the high SOD2 expression counterparts.

Conclusions: The SOD2 V16A variant may be associated with increased urological cancer susceptibility, especially for prostate cancer.

Methods: A pooled analysis utilizing odds ratios (ORs), *in silico* tools and ELISA was adopted to demonstrate this association. We also used immunohistochemical staining (IHS) to assess SOD2 expression.

## INTRODUCTION

Malignant tumor is considered as one of the leading causes of death worldwide, and it is estimated that more than 20 million new cases will be diagnosed each year by 2025 [1]. Despite the tremendous efforts made in the treatment of cancer, this disease still poses a serious threat to human health. Most of the malignancies result from 2 to 8 sequential changes, and single-base substitution is involved in 95% of these mutations [2–3]. Reactive oxygen species (ROS) have been indicated to cause DNA damage and induce genetic lesions, which play a crucial role in initiating mutagenic activity and carcinogenesis [4]. Superoxide dismutase 2 (SOD2) is one of the key endogenous antioxidants, shown to participate in the process of defense against mitochondrial ROS, a major source of cellular ROS [5]. Previous studies have indicated that genetic variation in ROS-related genes, encoding these enzymes, may reduce or impair the regulation of enzyme activity and alter the detoxification of ROS [6].

The SOD2 gene, located at sub band 6q25 of chromosome 6, is a homotetramer containing 2 identical subunits. This single-copy gene can encode superoxide dismutase-2, whose expression is significantly regulated at the transcription, translation, and posttranslational levels in the process of carcinogenesis [7–9]. Previously, it has been shown that the substitution of T-to-C in SOD2 gene can lead to the change of amino acids from valine (Val) to alanine (Ala) [10]. In addition, accumulating studies have indicated a correlation between the SOD2 V16A variant and risk of cancer. Recently, this variant has been demonstrated to be involved in a number of malignant tumors, such as breast cancer [11–12], colorectal cancer [13], prostate cancer [14], cervical cancer [15] and esophageal cancer [16]. The populations involved in the study of this genetic variant span over several ethnicities, such as Japanese [17], Americans [18], Brazilians [19], Egyptian [20], Turkish [21], and Italian [22].

Previous studies have investigated the correlation between SOD2 V16A variant and the risk of cancer, with some of these reports indicating the correlation of this polymorphism with higher cancer risk [11–14]. Nevertheless, some other researches did not indicate positive relationship between this variant and cancer risk [23–25]. Our current study is aimed at comprehensively estimating the possible association between SOD2 V16A variant and urological cancer risk [17–22, 26–45]. As the incidence of prostate cancer (PCa) is associated with aging phenomenon [46], we further used enzyme linked immunosorbent assay (ELISA) and immunohistochemical staining (IHS) to explore the expression of SOD2 among PCa participants enrolled in our centers.

## RESULTS

### Characteristics of our study

**Figure 1:**
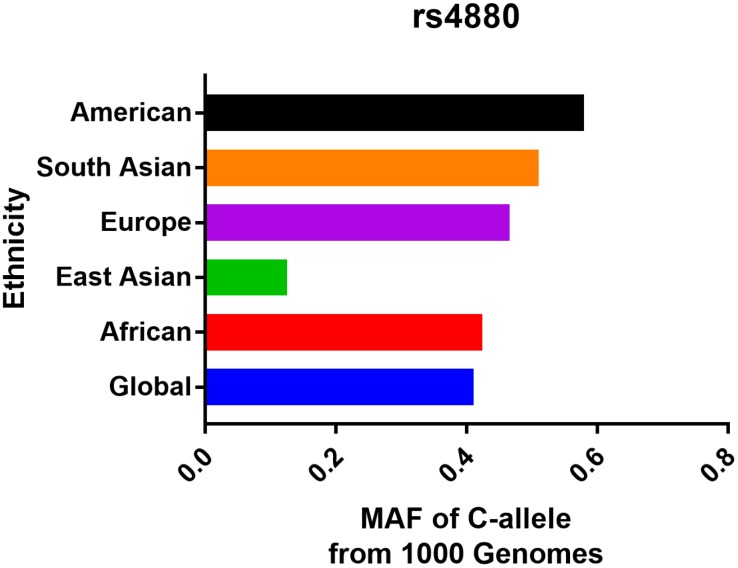
**Minor allele frequency for SOD2 rs4880 V16A variant in the main populations around the world.** Vertical line, ethnicity; Horizontal line, allele frequency.

As described in Supplementary Table 1, a total of 26 articles containing 28 case-control studies for investigating SOD2 V16A variant, were considered. Overall, 9,910 cancer cases and 11,239 controls were summarized. In subgroup analysis by ethnicity, 21 studies of these were based on Caucasian descendants, four studies were in African population, two were according to mixed population and only one was in Asian descendants. In stratified analysis by cancer type, 19 studies were investigating prostate cancer, 8 were based on bladder cancer, and one study was assessing renal cell carcinoma. 13 studies were performed utilizing hospital-based controls and 15 studies were population-based. Genotype distribution in control group was consistent with Hardy-Weinberg equilibrium (HWE) in 24 of the eligible studies. Moreover, we examined the minor allele frequency (MAF) of SOD2 V16A variant reported for the main populations around the world. For African descendants: A-allele (C) =0.424, V-allele (T) =0.576; for American population: A-allele = 0.580, V-allele = 0.420; for East Asian population: A-allele = 0.125, V-allele = 0.875; for South Asian: A-allele = 0.510, V-allele = 0.490; for European: A-allele = 0.466, V-allele = 0.534; for Global population: A-allele = 0.411, V-allele = 0.589 (Figure 1).

### Quantitative synthesis

**Figure 2:**
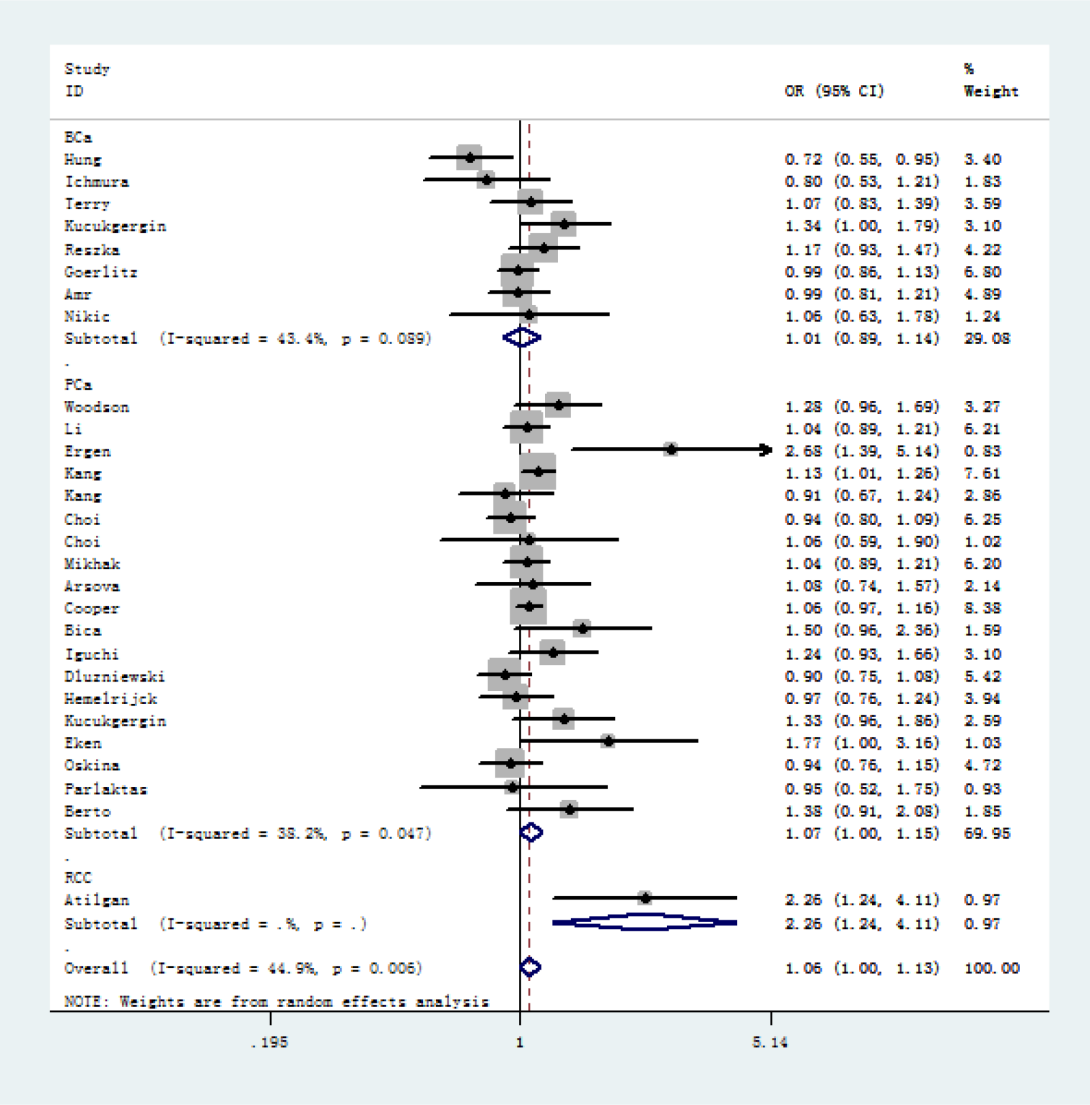
**Forest plot of cancer susceptibility correlated with SOD2 rs4880 V16A polymorphism (allelic comparison of A-allele vs.** V-allele, random-effects) in stratified analysis by the type of cancer.

**Figure 3:**
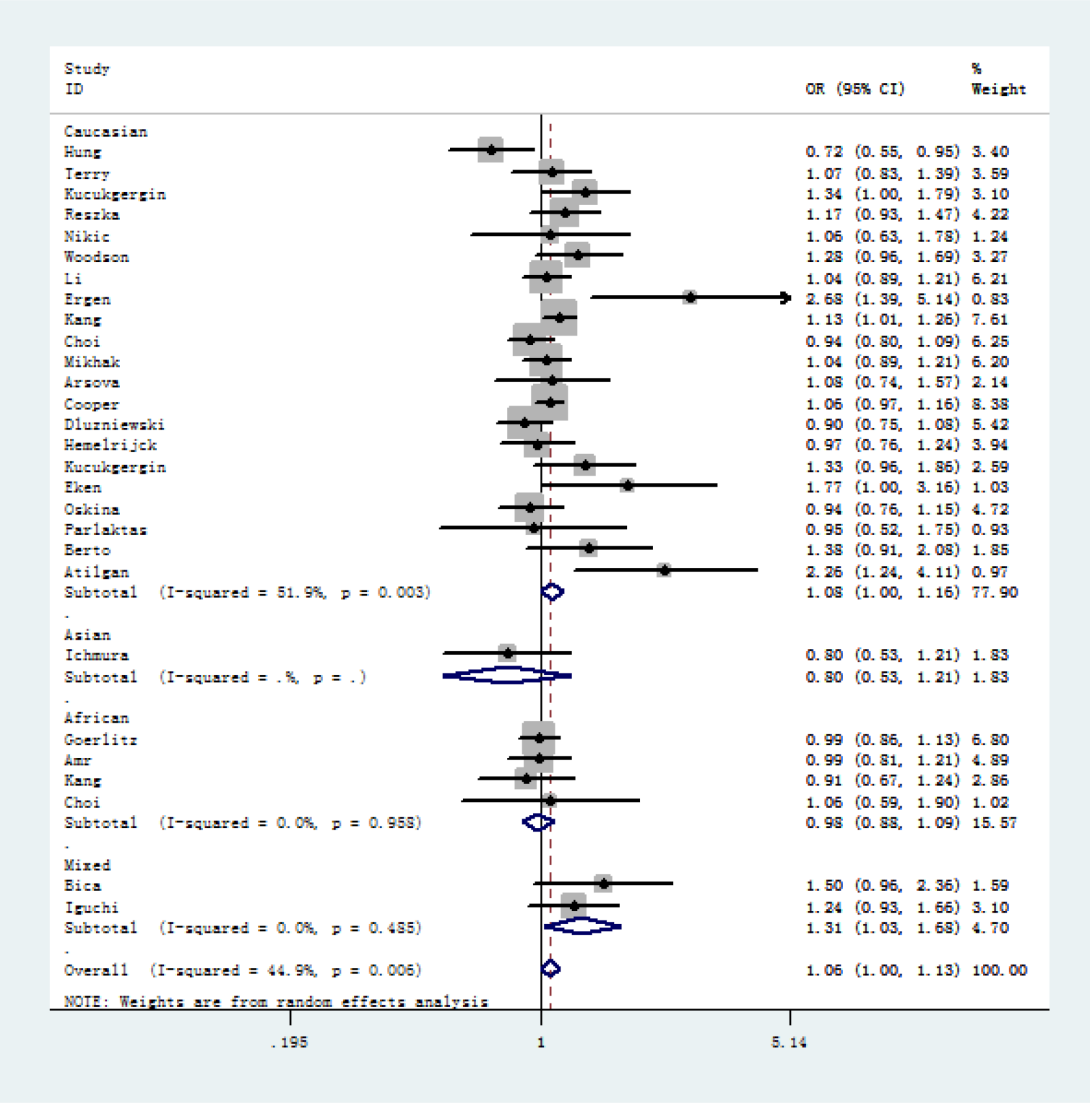
**Forest plot of A-allele versus V-allele genetic model of SOD2 rs4880 V16A polymorphism in stratified analysis by ethnicity (random-effects).**

In overall analysis, we identified a significant association between SOD2 V16A variant and urological cancer risk (A-allele vs. V-allele: OR = 1.06, 95% CI = 1.00 – 1.13, *P*_heterogeneity_ = 0.006, *P* = 0.047; AA+AV vs. VV: OR = 1.09, 95% CI = 1.02 – 1.16, *P* value for heterogeneity = 0.086, *P* = 0.008) (Table 2). In stratified analysis by cancer type, our results showed evidence that SOD2 V16A polymorphism is significantly associated with increased risk of prostate cancer (A-allele vs. V-allele: OR = 1.07, 95% CI = 1.00 – 1.15, *P*_heterogeneity_ = 0.047, *P* = 0.043, Figure 2; AA+AV vs. VV: OR = 1.12, 95% CI = 1.04 – 1.20, *P*_heterogeneity_ = 0.470, *P* = 0.003), but not for bladder cancer (A-allele vs. V-allele: OR = 1.01, 95% CI = 0.93 – 1.09, *P*_heterogeneity_ = 0.089, *P* = 0.892, Figure 2; AA+AV vs. VV: OR = 1.12, 95% CI = 1.04 – 1.20, *P*_heterogeneity_ = 0.470, *P* = 0.003). Moreover, in stratified analysis by race, we demonstrated positive correlation in Caucasian descendants (allele contrast: OR = 1.08, 95% CI = 1.00 – 1.16, *P*_heterogeneity_ = 0.003, *P* = 0.043, Figure 3; dominant comparison: OR = 1.11, 95% CI = 1.00 – 1.24, *P* value for heterogeneity = 0.034, *P* = 0.046). No obvious association was found in African (allele contrast: OR = 0.98, 95% CI = 0.88 – 1.09, *P*_heterogeneity_ = 0.958, *P* = 0.706; dominant comparison: OR = 1.01, 95% CI = 0.80 – 1.18, *P* value for heterogeneity = 0.908, *P* = 0.931) and Asian populations (allele contrast: OR = 0.80, 95% CI = 0.53 – 1.21, *P* = 0.295; dominant comparison: OR = 0.79, 95% CI = 0.50 – 1.24, *P* = 0.301). In stratified analysis by *P* value of HWE, we observed positive findings in studies that are consistent with HWE (allele contrast: OR = 1.05, 95% CI = 1.00 – 1.09, *P*_heterogeneity_ = 0.006, *P* = 0.031; dominant comparison: OR = 1.09, 95% CI = 1.02 – 1.17, *P* value for heterogeneity = 0.048, *P* = 0.010). Similarly, positive finding was indicated in studies with hospital-based controls (A-allele vs. V-allele: OR = 1.21, 95% CI = 1.02 – 1.43, *P*_heterogeneity_ = 0.001, *P* = 0.027; AV vs. VV: OR = 1.19, 95% CI = 1.01 – 1.39, *P* value for heterogeneity = 0.060, *P* = 0.038; AA vs. VV: OR = 1.40, 95% CI = 1.00 – 1.95, *P* value for heterogeneity = 0.002, *P* = 0.047; AA vs. AV+VV: OR = 1.32, 95% CI = 1.02 – 1.71, *P* value for heterogeneity = 0.009, *P* = 0.034).

**Table 2:** Stratified analyses of SOD rs4880 V16A polymorphism on urological cancer risk.

**Variables**	**N**	**Case/Control**	**OR(95%CI) *P*_heter_*P* A-allele vs. V-allele**	**OR(95%CI) *P*_heter_*P* AV vs. VV**	**OR(95%CI) *P*_heter_*P* AA vs. VV**	**OR(95%CI) *P*_heter_*P* AA+AV vs. VV**	**OR(95%CI) *P*_heter_*P* AA vs. AV+VV**
Total	28	9910/11239	1.06(1.00-1.13) 0.006 0.047	1.05(0.90-1.08) 0.031 0.359	1.13(1.00-1.28) 0.007 0.052	1.09(1.02-1.16) 0.086 0.008	1.08(0.97-1.20) 0.006 0.138
Ethnicity							
Caucasian	21	8020/9025	1.08(1.00-1.16) 0.003 0.043	1.01(0.94-1.09) 0.084 0.729	1.15(0.99-1.33) 0.008 0.060	1.11(1.00-1.24) 0.034 0.046	1.08(0.97-1.21) 0.017 0.175
African	4	1439/1675	0.98(0.88-1.09) 0.958 0.706	0.93(0.77-1.12) 0.596 0.417	0.95(0.78-1.17) 0.902 0.648	1.01(0.80-1.18) 0.908 0.931	0.94(0.79-1.12) 0.718 0.467
Mixed	2	238/330	1.31(1.03-1.68) 0.485 0.030	1.96(1.25-3.07) 0.190 0.003	1.87(1.11-3.17) 0.088 0.019	1.18(0.78-1.78) 0.434 0.436	1.89(1.23-2.90) 0.126 0.003
Asian	1	213/209	0.80(0.53-1.21) - 0.295	0.88(0.19-4.05) - 0.870	0.70(0.15-3.16) - 0.640	0.79(0.50-1.24) - 0.301	0.73(0.16-3.31) - 0.686
Cancer							
PCa	19	7478/8594	1.07(1.00-1.15) 0.047 0.043	1.06(0.92-1.23) 0.003 0.387	1.15(0.99-1.33) 0.022 0.064	1.12(1.04-1.20) 0.470 0.003	1.10(0.96-1.26) 0.002 0.177
BCa	8	2391/2595	1.01(0.93-1.09) 0.089 0.892	1.02(0.88-1.18) 0.922 0.782	1.03(0.87-1.21) 0.154 0.754	0.99(0.88-1.13) 0.059 0.930	1.02(0.89-1.17) 0.668 0.745
RCC	1	41/50	2.26(1.24-4.11) - 0.008	1.96(0.66-5.80) - 0.227	4.03(1.28-12.62) - 0.017	2.64(1.07-6.52) - 0.035	2.72(1.01-7.36) - 0.048
*P*_HWE_							
HWE	24	8665/9669	1.05(1.00-1.09) 0.006 0.031	1.00(0.93-1.08) 0.401 0.991	1.09(0.96-1.24) 0.043 0.167	1.09(1.02-1.17) 0.048 0.010	1.04(0.95-1.14) 0.136 0.432
non-HWE	4	1245/1570	1.07(0.96-1.20) 0.136 0.200	1.72(0.95-3.11) 0.001 0.072	1.70(0.96-3.01) 0.007 0.071	1.06(0.90-1.26) 0.564 0.495	1.73(0.98-3.04) 0.001 0.058
Source						
HB	13	1742/2450	1.21(1.02-1.43) 0.001 0.027	1.19(1.01-1.39) 0.060 0.038	1.40(1.00-1.95) 0.002 0.047	1.20(0.95-1.52) 0.005 0.129	1.32(1.02-1.71) 0.009 0.034
PB	15	8168/9152	1.04(0.99-1.08) 0.526 0.101	0.98(0.91-1.06) 0.222 0.637	1.08(0.99-1.18) 0.329 0.081	1.09(1.01-1.17) 0.838 0.022	1.01(0.94-1.09) 0.182 0.718

### Serum and tissue expression of SOD2

**Figure 4:**
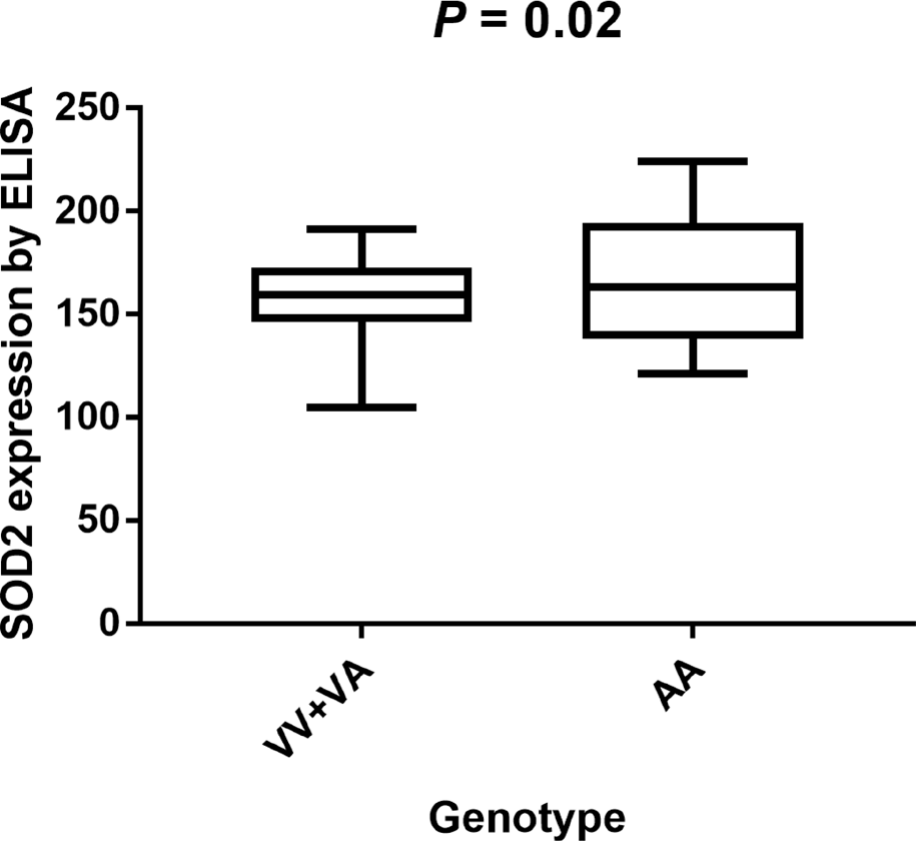
**Analysis of serum SOD2 levels in V16A genotype of PCa volunteers with mean values.** Serum SOD2 level of PCa patients with VV+VA genotypes was relatively lower than in those with AA genotypes (*P* = 0.02).

**Figure 5:**
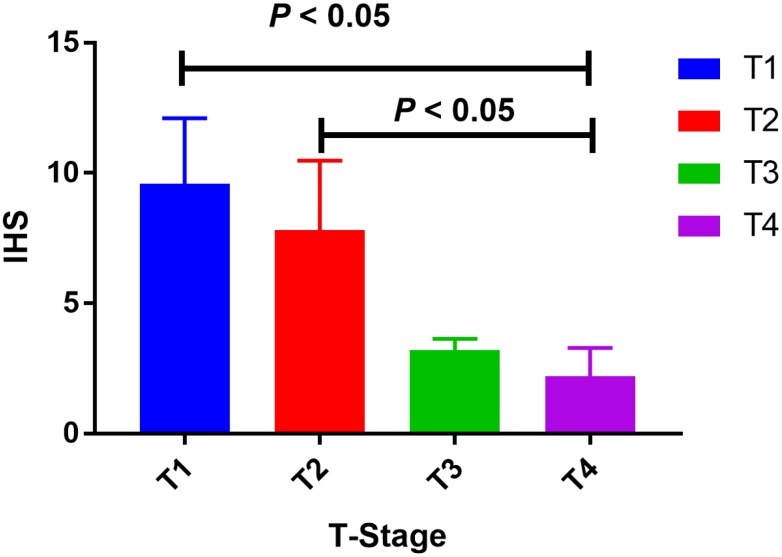
**Tissue expression of SOD2 among PCa subjects.** The expression of SOD2 was down-regulated in more advanced PCa, as compared to less advanced PCa subjects (T4 versus T1, *P* < 0.05; T4 versus T2*, P* < 0.05).

220 PCa patients’ serum samples were collected from various genotypes of SOD2 V16A polymorphism for our study. Moreover, the allele frequency of SOD2 V16A variant was also investigated. Allele distribution among the cancer patients enrolled in our centers was: AA, 67 (30.5%); AV, 40 (18.2%); VV 113(51.3%). Also, the MAF of SOD2 V16A variant was 0.270, slightly higher than that demonstrated in East Asian population (0.125), and lower than the MAF identified in South Asian population (0.510). Further, we utilized ELISA to evaluate the serum expression of SOD2 in our study population. Serum SOD2 level of PCa patients with VV+VA genotypes was relatively lower than in those with AA genotypes (Figure 4, *P* = 0.02). In order to corroborate with the expression of SOD2 in PCa tissues, we utilized IHS to test its expression among cancer subjects in our centers. As shown in Figure 5, the expression of SOD2 was downregulated in more advanced PCa, as compared to less advanced PCa subjects (T4 versus T1, *P* < 0.05; T4 versus T2*, P* < 0.05).

### *In silico* analysis

**Figure 6:**
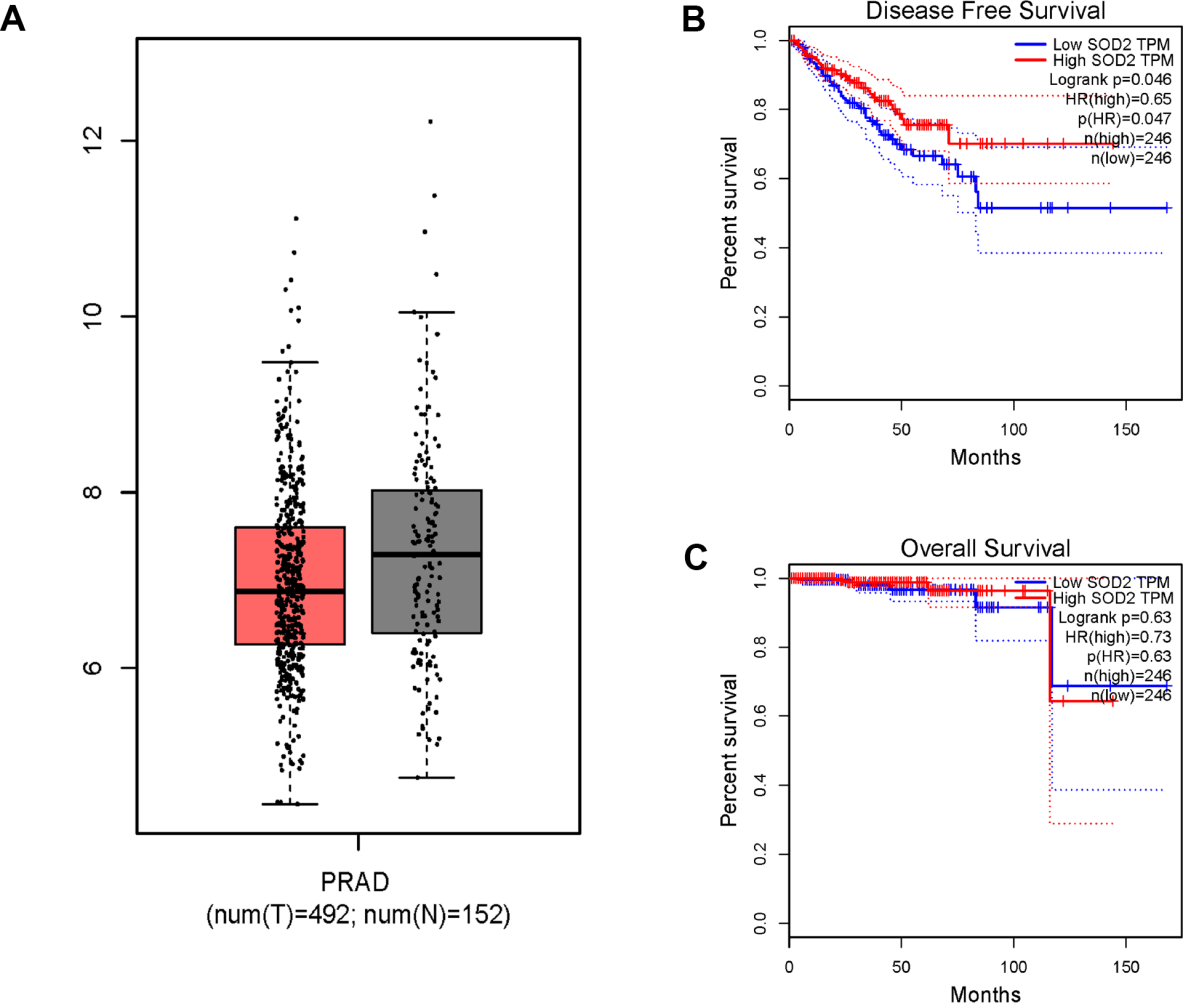
*In silico* analysis of SOD2 expression in prostate cancer patients (**A**), the investigation of disease-free survival (DFS) time (**B**) and overall survival (OS) time (**C**). Expression of SOD2 was down-regulated in prostate cancer tissues (Figure 6A, *P* < 0.05). Prostate cancer subjects with low SOD2 expression had a shorter DFS time than high SOD2 expression counterpart (Figure 6B, *P* = 0.047). No positive association was indicated for prostate cancer participants (Figure 6C, *P* = 0.630).

**Figure 7:**
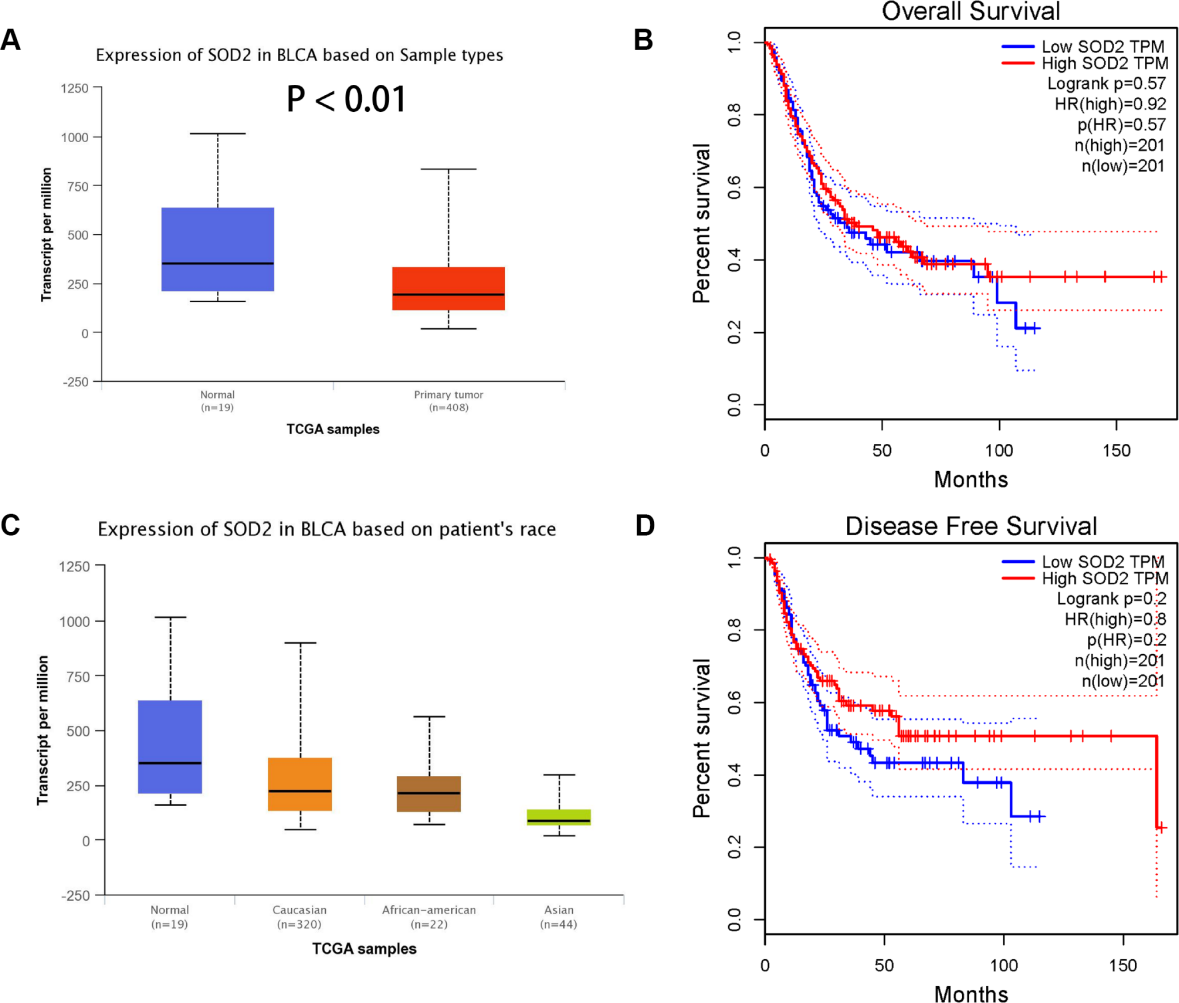
Association of SOD2 expression in bladder cancer subjects (**A**), based on patients’ race (**C**), the investigation of OS time (**B**) and DFS time (**D**). Expression of SOD2 was also down-regulated in bladder cancer tissues (Figure 7A), especially in Asian populations (Figure 7C, *P* < 0.05). No obvious difference was indicated in the effect of low SOD expression group and high expression group on OS time (Figure 7B, *P* = 0.570) and DFS time (Figure 7D, *P* = 0.200).

**Figure 8:**
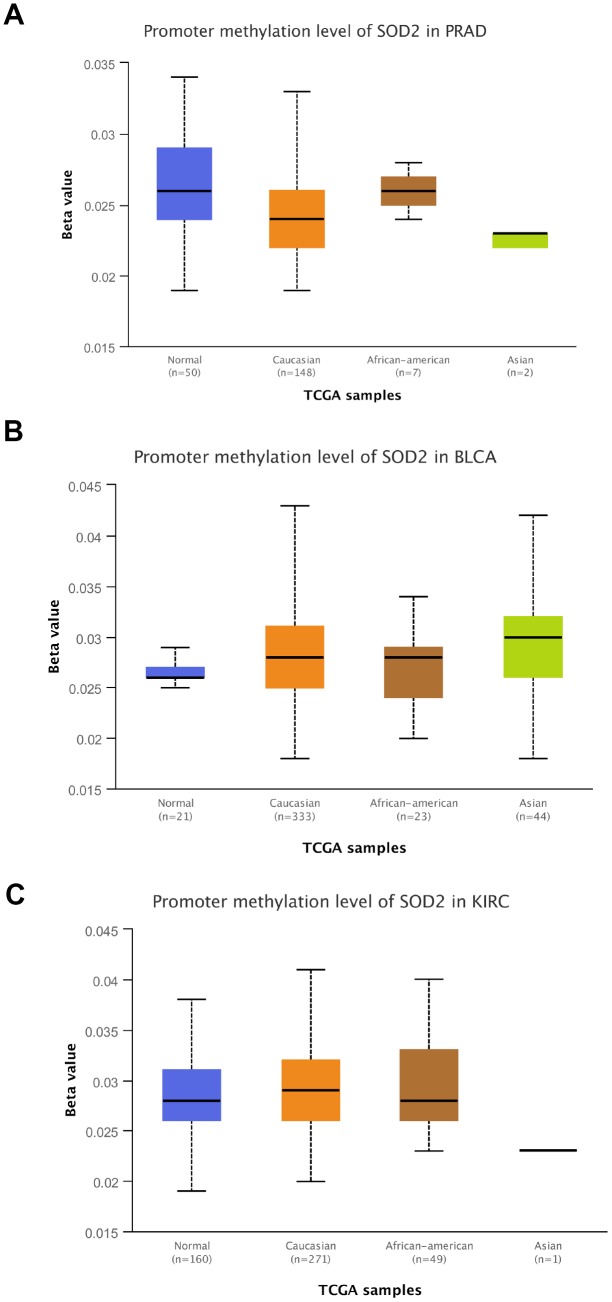
**Promoter methylation level of SOD2.** Promoter methylation level of SOD2 was decreased in both Caucasian and Asian prostate cancer participants (**A**). SOD2 promoter methylation level was both up-regulated in bladder cancer subjects (**B**). The methylation level was increased in Caucasian renal cell carcinoma patients and decreased in Asian cases (**C**).

**Figure 9:**
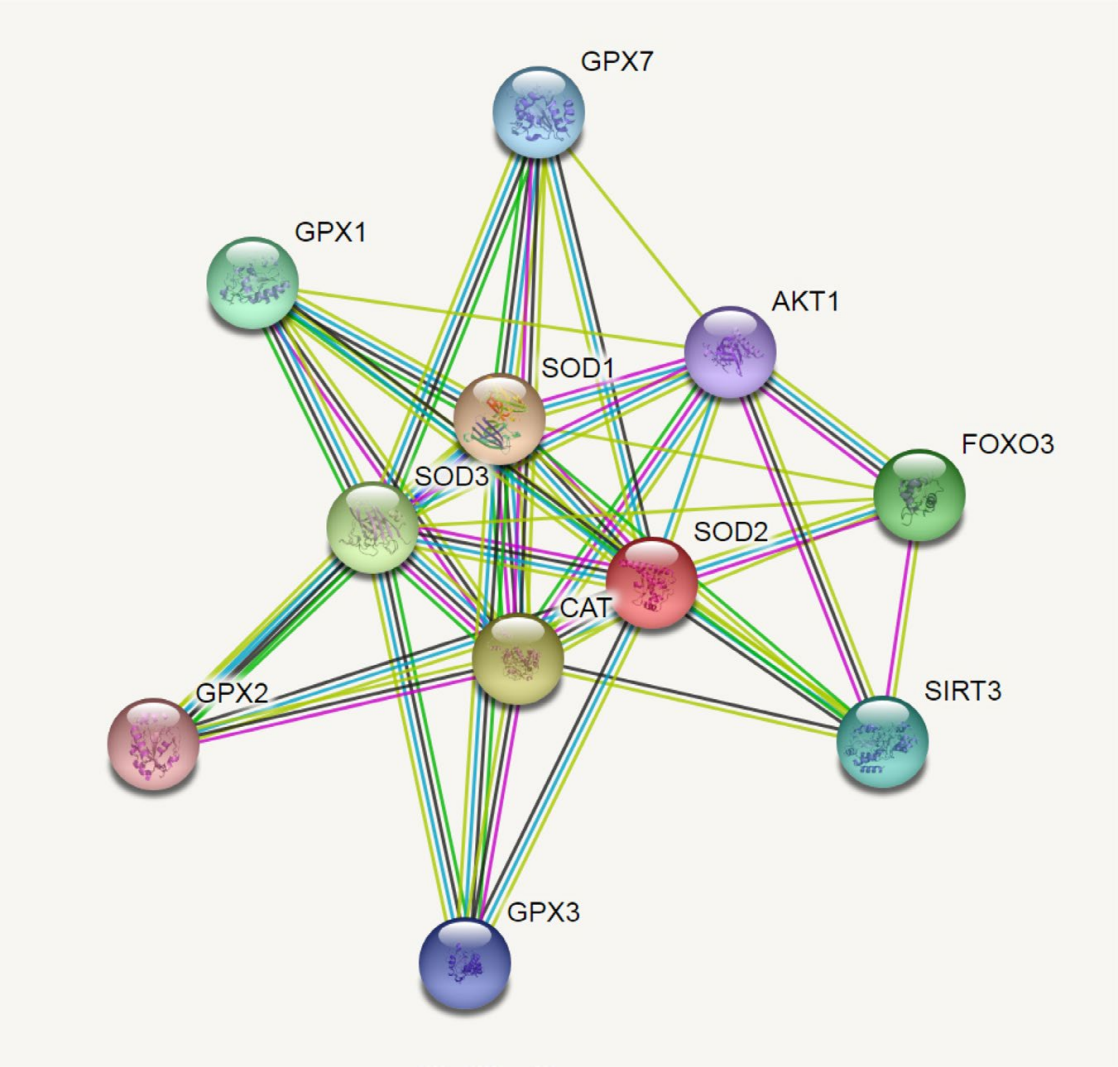
**SOD2 correlations crosstalk investigation by String server functional protein association networks (Homo sapiens).** At least 10 proteins were predicted to be involved in the interaction of SOD2, including SOD1 (Superoxide dismutase-1), CAT (Catalase), SOD3 (Extracellular superoxide dismutase-3), FOXO3 (Forkhead box protein O-3), GPX1 (Glutathione peroxidase 1), SIRT3 (NAD-dependent protein deacetylase sirtuin-3), GPX7 (Glutathione peroxidase-7), GPX3 (Glutathione peroxidase-3), AKT1 (RAC-alpha serine/threonine-protein kinase-1), GPX2 (Glutathione peroxidase-2).

**Figure 10:**
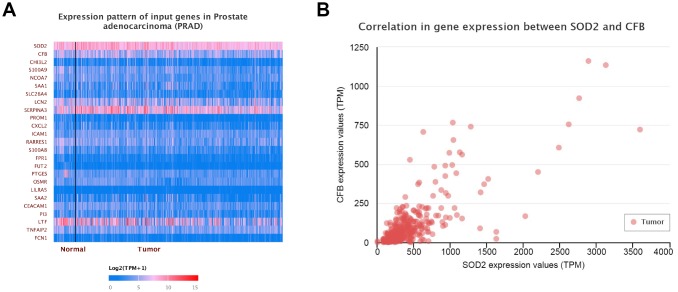
**Gene-gene interaction of SOD2.** At least 24 genes could participate in the correlation of SOD2 (**A**). Complement factor B gene (CFB) was predicted to be the most related gene. There was a positive correlation between CFB and SOD2 in PCa (**B**).

**Figure 11:**
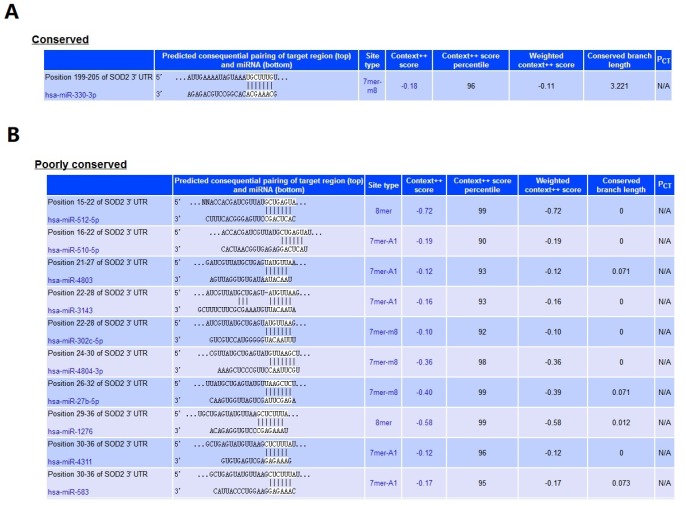
**MiRNA that related to SOD2.** At least 11 miRNA were predicted to be related to SOD2 by TargetScan database. The hsa-miR-330-3p (**A**) was highly conserved, and the rest ten were poorly conserved (**B**).

**Figure 12:**
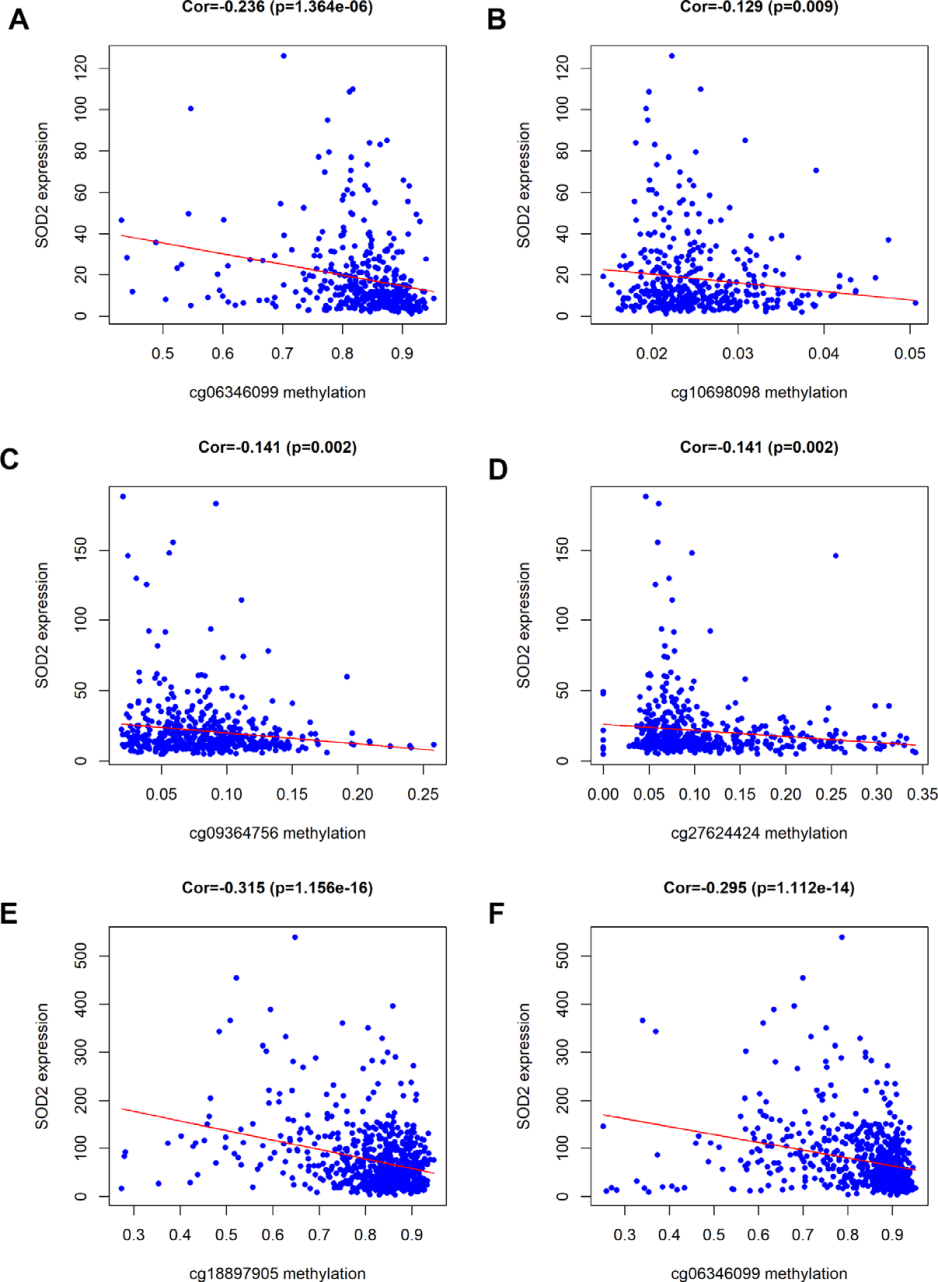
**Association of DNA methylation and SOD2 expression.** According to the analysis of TCGA data, SOD2 expression was negatively associated with the levels of methylation at six CpG sites (cg06346099 and cg10698098 for BCa, cg09364756 and cg27624424 for PCa, cg18897905 and cg06346099 for RCC).

Results from *in silico* tools showed that the expression of SOD2 is downregulated in both prostate (Figure 6A) and bladder cancer tissues (Figure 7A). Expression of SOD2 was especially decreased in Asian bladder cancer subjects (Figure 7C, *P* < 0.05). In addition, prostate cancer subjects with low SOD2 expression had a shorter DFS time than high-SOD2-expression counterparts (Figure 6B, *P* = 0.047). No positive finding was observed for bladder cancer (Figure 7D, *P* = 0.200). Moreover, the relationship between the expression of SOD2 and overall survival time of prostate and bladder cancer was also investigated by Kaplan-Meier estimate. Unfortunately, no positive association was indicated for either prostate (Figure 6C, *P* = 0.630) or bladder cancer (Figure 7B, *P* = 0.570). The Cancer Genome Atlas (TCGA) samples were utilized to investigate the level of promoter methylation for SOD2 gene in different urological cancers. The promoter methylation level of SOD2 was found to be decreased in both Caucasian and Asian prostate cancer participants (Figure 8A). Nevertheless, SOD2 promoter methylation level was upregulated in bladder cancer subjects (Figure 8B). Additionally, the methylation level was increased in Caucasian renal cell carcinoma patients and decreased in the Asian cases (Figure 8C). Furthermore, we used String online tool to evaluate the functional protein association of SOD2 (http://string-db.org/). As described in Figure 9, more than 10 proteins were predicted to be involved in the interaction of SOD2, including SOD1 (Superoxide dismutase-1), CAT (Catalase), SOD3 (Extracellular superoxide dismutase- 3), FOXO3 (Forkhead box protein O-3), GPX1 (Glutathione peroxidase 1), SIRT3 (NAD-dependent protein deacetylase sirtuin-3), GPX7 (Glutathione peroxidase-7), GPX3 (Glutathione peroxidase-3), AKT1 (RAC-alpha serine/threonine-protein kinase-1), GPX2 (Glutathione peroxidase-2). The gene-gene interaction of SOD2 among prostate cancer participants was also evaluated by TCGA samples. As described in Figure 10A, at least 24 genes were reported to participate in the correlation of SOD2. Among them, complement factor B gene (CFB) was predicted to be the most related gene in prostate cancer. There was a positive correlation between them in prostate cancer (Figure 10B). As was shown in Figure 11, at least 11 miRNA were predicted to be related to SOD2 by TargetScan database. The hsa-miR-330-3p was highly conserved miRNA (Figure11 A), and the rest ten were poorly conserved (Figure11B). To evaluate the correlation of DNA methylation and SOD2 expression, we adopted scatter plots to investigate the relationship between CpG sites and SOD2 expression based on three urological cancers (bladder cancer, prostate cancer, and renal cell carcinoma) in TCGA database. For bladder cancer, SOD2 expression was negatively associated with methylation levels at two CpG sites (cg06346099 and cg10698098, *P* < 0.05, Figure12A and 12B). The cg09364756 and cg27624424 methylation were correlated with SOD2 expression in prostate cancer (*P* < 0.05, Figure12C and 12D). For renal cell carcinoma, SOD2 expression was negatively associated with cg18897905 and cg06346099 methylation (*P* < 0.05, Figure 12E and 12F).

### Publication bias and sensitivity analyses

**Figure 13:**
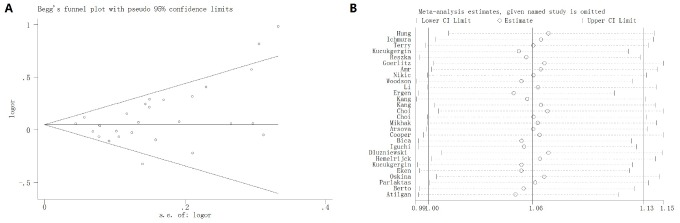
Begg’s funnel plot (**A**) and sensitivity analysis (**B**) for SOD2 rs4880 V16A polymorphism under allelic contrast model. No evidence of publication bias was identified for SOD2 V16A variant by Begg’s funnel plots test (t = 2.17, *P* = 0.119). No single study could have an impact on the pooled OR through sensitivity analysis.

Egger’s test and Begg’s plot were utilized to investigate any publication bias in the enrolled studies. No evidence of publication bias was identified for SOD2 V16A variant (A-allele versus V-allele: t = 2.17, *P* = 0.119; AV versus VV: t = 2.03, *P* = 0.173; AA versus VV: t = 2.06, *P* = 0.213; AA+VA vs. VV: t = 2.16, *P* = 0.110; AA vs. VA + VV: t = 2.03, *P* = 0.149, Figure 13A). Sensitivity analysis was also carried out to check the effect of each study on pooled ORs by repeating the meta-analysis when each time an individual study was removed. The sensitivity analysis for the relationship of SOD2 V16A variant in the allelic contrast is described in Figure 13B, indicating that no single study could have an impact on the pooled OR. These results suggested that conclusions drawn from the present analyses are reliable.

## DISCUSSION

Previous studies have shown that SOD plays a central role in protecting organisms from the harmful effets of superoxide free radicals, by converting them into hydrogen peroxide [5, 47]. Further in vivo experiments utilizing SOD2-deficient mice showed perinatal death, myocardial injury and neurodegeneration caused by impaired SOD2 activity [48, 49]. SOD2, as one of the most crucial enzymes against mitochondrial ROS, has also been found to act as a potential tumor suppressor gene in carcinogenesis [50, 51]. Some studies have shown that the activity and expression of SOD2 in cancer cells are significantly down regulated as compared to that in control cells [52, 53].

Till date, several studies have assessed the relationship between SOD2 V16A variant and cancer susceptibility; however, their conclusions remain inconsistent [17–22]. A previous study in Macedonian population indicated that SOD2 V16A variant is associated with risk of prostate cancer [33]. This finding was also confirmed by Kucukgergin et al based on Turkish descendants [38]. Nevertheless, Choi and his group indicated a different result [32]. Li et al [14] performed a meta-analysis and found that SOD2 V16A variant was associated with increased prostate cancer risk. Conversely, another meta-analysis conducted by Bag et al [54] indicated that this polymorphism was not significantly associated with overall cancer risk. Therefore, the overall objective of this study was to assess all eligible data on the basis of inclusion criteria in order to improve statistical effectiveness and acquire more reliable conclusions.

In the present study, a total of 9,910 cancer subjects and 11,239 control participants were accounted into the analysis. Overall results indicated that SOD Val16Ala polymorphism is correlated with increased urological cancer susceptibility, especially for prostate cancer, which is consistent with previous findings. [14, 33, 55]. In stratified analysis by race, we observed similar findings in Caucasians and mixed populations, but not in Asians and Africans. Stratification analysis also revealed that this correlation was more obvious in hospital-based and high quality studies. *In silico* tools showed evidence that the expression of SOD2 is downregulated in both prostate and bladder cancer tissues as compared to that in control. To verify this finding, we utilized ELISA to evaluate the serum expression of SOD2 in our study population and revealed that the serum SOD2 level in PCa patients with VV+VA genotypes was relatively lower than in those with AA genotypes. Besides, we utilized IHS to further investigate the expression of SOD2 in different stages of PCa cases and found that SOD2 expression was downregulated in more advanced PCa as compared to less advanced PCa subjects. Results from *in silico* tools indicated that the expression of SOD2 was downregulated in both prostate and bladder cancer tissues as compared to the control samples. Furthermore, prostate cancer subjects with low SOD2 expression had a shorter DFS time than the high-SOD2-expression counterpart. According to the analysis of TCGA data, SOD2 expression was negatively associated with the levels of methylation at six CpG sites (cg06346099 and cg10698098 for BCa, cg09364756 and cg27624424 for PCa, cg18897905 and cg06346099 for RCC).

It is important to consider the limitations of the current analysis which might have an influence on the final conclusion. First, the number of registered articles in the present analysis is still insufficient for a more comprehensive analysis. Only four studies were based on African population and one was towards Asian descendants. Second, subjects from hospitals or populations may have potential diseases, which may affect the health of participants and the findings of this study. In addition, we did not evaluate the serum SOD2 level in healthy participants due to ethical factors. In stratification analysis by cancer type, only one study was for renal cell carcinoma. We tried to further assess the potential interactions between SOD2 V16A variant and different stages and grades of tumors; however, the original data remains insufficient. As described in Figure 9, according to String analysis, at least ten proteins might participate in the interaction with SOD2. However, TCGA samples showed more than 24 genes to be correlated with SOD2 in prostate cancer. Complement factor B gene (CFB) was predicted to be the most related gene. However, there are few studies on the specific mechanism of CFB gene in prostate cancer. The hsa-miR-330-3p was predicted to be highly conserved miRNA related to SOD2. As no further investigation on their correlation could be identified from the online database, future *in vitro* and functional experiment are required to verify these interactions in more detail. Importantly, future research is still warranted to ascertain whether the SOD2 V16A variant is responsible for the reduced SOD2 gene expression. Moreover, some advantages of the present analysis need to be mentioned. First, all eligible studies that assessed the relationship between SOD2 V16A variant and urological cancer risk were enrolled in the current analysis, which could acquire more reliable conclusions compared to a single study. Besides, the Begg’s plot and Egger’s test demonstrated no evidence of publication bias, which indicated that the conclusions drawn from the present analyses are reliable.

## CONCLUSIONS

Taken together, the current analyses demonstrate that SOD2 V16A variant may be associated with increased susceptibility to urological cancer, especially for prostate cancer. Moreover, the expression of SOD2 was found to be downregulated in more advanced prostate cancer participants, as compared to the less advanced ones. Further high quality randomized controlled studies are necessary to ascertain the correlation between SOD2 V16A variant and urological cancer risk or survival in more detail.

## MATERIALS AND METHODS

### Search strategy

All suitable studies on SOD2 variant and cancer risk were retrieved by systematically searching databases including Embase, PubMed, Google scholar, Chinese National Knowledge Infrastructure (CNKI), and Wanfang databases (the last search was conducted on August 22, 2019). The search keywords were as follows: “SOD2” or “Superoxide Dismutase 2”, “variant” or “polymorphism”, “cancer” or “tumor” or “carcinoma”. Additional suitable publications were hand-searched from original studies or references about this topic.

### Inclusion and exclusion criteria

Two investigators selected case-control studies according to the following inclusion criteria: (a) studies compared cancer with control; (b) investigating the correlation between SOD2 V16A variant and urological cancer risk (including prostate cancer, bladder cancer and renal cell carcinoma); (c) providing sufficient genotype data and allele distribution for calculating odds ratio with 95% confidence interval. If any of the following aspects exist, the study was excluded: (a) without suitable genotype data; (b) studies without controls; (c) duplicate publications with previous data.

### Data extraction

Two authors independently reviewed and identified the eligible studies based on the criteria mentioned above. Detailed information of the extracted studies was as follows: first author’s name, publication year, ethnicity of study population, control source (hospital-based or population-based), type of cancer, total number of case and control with V/V, V/A, A/A genotypes, *P* value of Hardy-Weinberg equilibrium (HWE) in control, age range, method of genotyping. Controversial content should be addressed by discussion of all investigators to reach a final consensus.

### Statistical analysis

The strength of correlation between SOD2 V16A and urological cancer susceptibility was measured by odds ratios (ORs) combined with 95% confidence intervals (CIs). Pooled ORs of five comparison models were investigated: allelic comparison (A-allele versus V-allele), homozygous model (AA versus VV), heterozygous model (VA versus VV), dominant comparison (AA+VA vs. VV), and recessive comparison (AA vs. VA + VV). We employed Chi-square-based *Q* test to assess statistical heterogeneity among studies. If *P* value less than 0.05, heterogeneity was considered significant. Therefore, the fixed-effects model (Mantel-Haenszel method) was conducted. Otherwise, random-effects model (DerSimonian-Laird method) was adopted. Subgroup analyses were measured by ethnicity (Caucasian, Asian, African, or mixed population), type of cancer (prostate cancer, bladder cancer and renal cell carcinoma), source of control (hospital-based and population-based studies). Hardy-Weinberg equilibrium (HWE) in control group was also calculated. If *P* value of HWE less than 0.05, it should be defined as low quality study (Classified as non-HWE group). We applied Begg’s funnel plots and Egger’s test to check publication bias among studies. *P* value less than 0.05 can be defined as the existence of significant publication bias. Moreover, we applied sensitivity analysis to determine the stability of final result by omitting one study each time. STATA software (v11.0; Stata Corporation, TX) was employed in all of the above statistical analyses.

### Study population

Overall, 220 pathologically confirmed prostate cancer subjects were recruited from the Affiliated Changzhou No.2 People’s Hospital of Nanjing Medical University and Affiliated Hospital of Jiangnan University. Distribution of PCa patients’ characteristics was summarized in Table 1. These patients were diagnosed with prostate cancer through needle biopsy (from February 2013 to July 2018). 2 milliliters of peripheral blood samples were collected from every enrolled prostate cancer participants. Before all blood samples were prepared, written informed consent should be acquired from every study subjects. The present study protocol was approved by the above hospitals.

**Table 1:** Distribution of characteristics from the PCa patients involved in our hospitals.

**Features**	**PCa patients**
N	220
Age,n(%)	
<60	101(45.9)
≥60	119(54.1)
Smoking, n (%)	
Ever	99(45)
Never	121(55)
Alcohol drinking, n (%)	
Ever	131(59.5)
Never	89(40.5)
PSA, n (%)	
4-10	133(60.4)
10-20	69(31.3)
>20	18(8.3)
Gleason score (%)	
<7	101(45.9)
=7	69(31.4)
>7	50(22.7)
TNM stage (%)	
≤T2c	151(68.6)
=T3a	44(20)
≥T3b	25(11.4)
Recurrent (%)	
Yes	19 (8.6)
No	201(91.4)

### Genotyping methods

Genotyping of SOD2 V16A polymorphism was carried out using different techniques in various studies, such as real-time PCR, restriction fragment length polymorphism PCR (PCR-RFLP), MassArray (Sequenom, San Diego, CA), Mass spectrometry (matrix-assisted laser desorption/ionization time-of-flight) (Sequenom, San Diego, CA). In our experiment, SOD2 V16A polymorphism was determined using TaqMan assay by Li et al. [56]

### Enzyme Linked immunosorbent Assay (ELISA) and immunohistochemical staining (IHS)

Blood of participants was gathered in standard cubes without anticoagulant. We applied serum separator tube (SST) and solidified the sample at room temperature for 2 hours, and then centrifuged at 1000 × g for 15 minutes. Take out the serum immediately and determine it, and divide it equally or store the sample at -80 °C. Serum SOD2 expression of participants recruited from our centers was tested by ELISA kit (CUSABIO Co. ltd.). Moreover, we utilized IHS to test the tissue expression of SOD2 among PCa subjects in our centers. Paraffin section of prostate cancer was incubated in hydrogen peroxide (1%) and then washed in PBS. We used goat serum to block the binding of non-specific proteins. Then the slice was incubated with anti-SOD2 antibody at 1: 200. The immunoreactive sites were shown brown with diaminobenzidine.

### *In silico* analysis of SOD2 expression

We applied online gene expression database to evaluate SOD2 expression in prostate and bladder cancer based on different ethnic population (http://gemini.cancer-pku.cn/). We further adopted The Cancer Genome Atlas (TCGA) samples to evaluate high and low expression of SOD2 on overall survival time and BCa: bladder cancer; HWE: Hardy-Weinberg equilibrium; HB: hospital-based; PB: population-based; *P*_heter_: *P* value of *Q*-test for heterogeneity test; *P*_HWE_: *P* value of HWE; PCa: prostate cancer; RCC: renal cell carcinoma.

disease free survival time. Promoter methylation levels of SOD2 in different urological cancers were also evaluated. String online server was employed to investigate functional protein association of SOD2 (http://string-db.org/). We further utilized TCGA samples to investigate gene-gene interaction of SOD2 among prostate cancer participants (http://ualcan.path.uab.edu/analysis.html). Promoter methylation level of SOD2 was also investigated by TCGA samples (http://ualcan.path.uab.edu/cgi-bin/TCGA-methyl-Result.pl?genenam=SOD2). Additionally, TargetScan database was utilized to predict the possible miRNA correlated to SOD2 (http://www.targetscan.org/vert_71/).

## Supplementary Material

Supplementary Table 1
