# Supplementary material for: Association between SOD2 V16A variant and urological cancer risk
Source: Aging (Albany NY). 2020 Jan 12;12(1):825–43. doi: 10.18632/aging.102658 (PMC6977677; doi:10.18632/aging.102658)
Supplement: Supplementary Table 1 [file aging-12-102658-s001..doc]

**Supplementary Table 1. Basic information of included studies for SOD2 rs4880 V16A variant and urological cancer risk.**

| First author | Year | Origin | Cancer | Ethnicity | Source | Case | Control | Case |  |  | Control | |  | HWE | Age range |  | Method |
| --- | --- | --- | --- | --- | --- | --- | --- | --- | --- | --- | --- | --- | --- | --- | --- | --- | --- |
|  |  |  |  |  |  |  |  | AA | AV | VV | AA | AV | VV |  | Case | Control |  |
| Hung | 2004 | Italy | BCa | Caucasian | HB | 201 | 214 | 44 | 89 | 68 | 54 | 115 | 45 | 0.262 | 20-80 | 20-80 | PCR-RFLP |
| Ichmura | 2004 | Japan | BCa | Asian | PB | 213 | 209 | 3 | 41 | 169 | 4 | 48 | 157 | 0.882 | 65.82±12.2 | 57.53±8.68 | PCR |
| Terry | 2005 | USA | BCa | Caucasian | PB | 235 | 214 | 59 | 122 | 54 | 54 | 103 | 57 | 0.586 | 65.7±10.7 | 63.3±10.3 | MS |
| Kucukgergin | 2012 | Turkey | BCa | Caucasian | HB | 157 | 224 | 37 | 68 | 52 | 36 | 99 | 89 | 0.341 | 63.2±10.86 | 61.7±8.39 | PCR |
| Reszka | 2014 | Poland | BCa | Caucasian | HB | 244 | 364 | 74 | 110 | 60 | 98 | 161 | 105 | 0.028 | 66.5±10.5 | 61.3±10.4 | RT-PCR |
| Goerlitz | 2014 | Egypt | BCa | African | PB | 894 | 802 | 209 | 413 | 272 | 191 | 369 | 242 | 0.031 | 58.5±10.8 | 56.4±12.0 | TaqMan |
| Amr | 2015 | Egypt | BCa | African | PB | 414 | 356 | 99 | 188 | 127 | 87 | 160 | 109 | 0.065 | 59.6±10.4 | 58.5±11.7 | TaqMan |
| Nikic | 2018 | Serbia | BCa | Caucasian | HB | 33 | 212 | 7 | 17 | 9 | 47 | 99 | 66 | 0.395 | 65.45±9.07 | 63.42±7.97 | PCR-RFLP |
| Woodson | 2003 | USA | PCa | Caucasian | PB | 199 | 191 | 58 | 98 | 43 | 40 | 102 | 49 | 0.330 | 60.8±5.2 | 60.6±5.0 | MS |
| Li | 2005 | USA | PCa | Caucasian | PB | 567 | 764 | 147 | 288 | 132 | 195 | 379 | 190 | 0.829 | 60.7±7.6 | 60.8±7.6 | PCR-RFLP |
| Ergen | 2007 | Turkey | PCa | Caucasian | HB | 50 | 50 | 6 | 25 | 19 | 0 | 18 | 32 | 0.121 | 68.11±9.13 | 64.36±8.68 | PCR-RFLP |
| Kang | 2007 | USA | PCa | Caucasian | PB | 1150 | 1382 | 297 | 578 | 275 | 320 | 686 | 376 | 0.835 | 64.8±5.0 | 64.5±5.0 | TaqMan |
| Kang | 2007 | USA | PCa | African | PB | 103 | 395 | 15 | 57 | 31 | 79 | 194 | 122 | 0.906 | 64.8±5.0 | 64.5±5.0 | TaqMan |
| Choi | 2007 | USA | PCa | Caucasian | PB | 455 | 1214 | 104 | 239 | 112 | 311 | 610 | 293 | 0.857 | NA | NA | MS |
| Choi | 2007 | USA | PCa | African | PB | 28 | 122 | 6 | 15 | 7 | 31 | 52 | 39 | 0.112 | NA | NA | MS |
| Mikhak | 2008 | USA | PCa | Caucasian | PB | 642 | 652 | 166 | 320 | 156 | 159 | 331 | 162 | 0.695 | 66.0±7.0 | 66.0±7.0 | TaqMan |
| Arsova | 2008 | Turkey | PCa | Caucasian | HB | 85 | 151 | 20 | 46 | 19 | 37 | 73 | 41 | 0.690 | 69.4±7.6 | 69.2±7.7 | RT-PCR |
| Cooper | 2008 | USA | PCa | Caucasian | PB | 2634 | 1636 | 680 | 1352 | 602 | 424 | 789 | 423 | 0.152 | NA | NA | TaqMan |
| Bica | 2009 | Brazil | PCa | Mixed | PB | 51 | 155 | 9 | 32 | 10 | 8 | 105 | 42 | <0.001 | 52.62-75.88 | 56.64-71.87 | PCR-RFLP |
| Iguchi | 2009 | USA | PCa | Mixed | HB | 187 | 175 | 60 | 86 | 41 | 39 | 96 | 40 | 0.199 | 63.3±8.0 | 62.3±9.9 | PCR-RFLP |
| Dluzniewski | 2012 | USA | PCa | Caucasian | HB | 472 | 472 | 108 | 233 | 131 | 119 | 236 | 117 | 0.999 | 58.9±6.2 | 59.0±5.9 | MassArray |
| Hemelrijck | 2012 | Germany | PCa | Caucasian | PB | 203 | 360 | 53 | 100 | 50 | 90 | 190 | 80 | 0.285 | 57.91±5.0 | 57.93±5.0 | MassArray |
| Kucukgergin | 2012 | Turkey | PCa | Caucasian | HB | 134 | 159 | 26 | 65 | 43 | 24 | 69 | 66 | 0.398 | 64.1±7.48 | 62.5±7.53 | PCR-RFLP |
| Eken | 2013 | Turkey | PCa | Caucasian | HB | 33 | 81 | 9 | 17 | 7 | 13 | 37 | 31 | 0.726 | 67.52±9.31 | 64.63±8.94 | RT-PCR |
| Oskina | 2014 | Russia | PCa | Caucasian | PB | 380 | 337 | 94 | 194 | 92 | 99 | 152 | 86 | 0.076 | 69.2±8.4 | 64.3±14.7 | TaqMan |
| Parlaktas | 2015 | Turkey | PCa | Caucasian | HB | 49 | 49 | 3 | 23 | 23 | 5 | 20 | 24 | 0.784 | 64.22±7.77 | 63.27±8.08 | Probe |
| Berto | 2015 | Brazil | PCa | Caucasian | HB | 56 | 249 | 13 | 28 | 15 | 18 | 165 | 66 | <0.001 | 66.47±7.75 | 64.27±7.79 | PCR |
| Atilgan | 2014 | Turkey | RCC | Caucasian | HB | 41 | 50 | 14 | 17 | 10 | 8 | 19 | 23 | 0.244 | 59.54±12.96 | 62.18±8.44 | Probe |

BCa: bladder cancer; HB: hospital-based; HWE: Hardy-Weinberg equilibrium of controls; MS: Mass spectrometry; NA: not available; PB: population-based; PCa: prostate cancer; PCR-RFLP: polymerase chain reaction and restrictive fragment length polymorphism; RCC: renal cell carcinoma; RT: real time.
